# A novel, efficient method for estimating the prevalence of acute malnutrition in resource-constrained and crisis-affected settings: A simulation study

**DOI:** 10.1371/journal.pone.0186328

**Published:** 2017-11-01

**Authors:** Severine Frison, Marko Kerac, Francesco Checchi, Jennifer Nicholas

**Affiliations:** Department of Population Health, London School of Hygiene and Tropical Medicine (LSHTM), London, United Kingdom; The Hospital for Sick Children, CANADA

## Abstract

**Introduction:**

The assessment of the prevalence of acute malnutrition in children under five is widely used for the detection of emergencies, planning interventions, advocacy, and monitoring and evaluation. This study examined PROBIT Methods which convert parameters (mean and standard deviation (SD)) of a normally distributed variable to a cumulative probability below any cut-off to estimate acute malnutrition in children under five using Middle-Upper Arm Circumference (MUAC).

**Methods:**

We assessed the performance of: PROBIT Method I, with mean MUAC from the survey sample and MUAC SD from a database of previous surveys; and PROBIT Method II, with mean and SD of MUAC observed in the survey sample. Specifically, we generated sub-samples from 852 survey datasets, simulating 100 surveys for eight sample sizes. Overall the methods were tested on 681 600 simulated surveys.

**Results:**

PROBIT methods relying on sample sizes as small as 50 had better performance than the classic method for estimating and classifying the prevalence of acute malnutrition. They had better precision in the estimation of acute malnutrition for all sample sizes and better coverage for smaller sample sizes, while having relatively little bias. They classified situations accurately for a threshold of 5% acute malnutrition. Both PROBIT methods had similar outcomes.

**Conclusions:**

PROBIT Methods have a clear advantage in the assessment of acute malnutrition prevalence based on MUAC, compared to the classic method. Their use would require much lower sample sizes, thus enable great time and resource savings and permit timely and/or locally relevant prevalence estimates of acute malnutrition for a swift and well-targeted response.

## Introduction

Acute malnutrition (AM) is a major public health issue throughout low-middle income countries. Indices of AM include low Weight-for-Height/Length (WFH), low Middle-Upper-Arm Circumference (MUAC) and oedmatous malnutrition (characterised by the presence of bilateral pitting oedema) (see Table 1). The United Nations Children's Fund’s (UNICEF) latest report on the State of the World’s Children [1] estimates that 9% of children under 5 years old in least developed countries have a low WFH. According to United Nations estimates 875 000 children under five deaths [2] are attributed to Low WFH annually. These estimates do not include oedematous malnutrition. Overall, prevalence estimates of Global Acute Malnutrition (GAM) are similar whether including oedematous malnutrition or not [3]. There is an increasing interest in MUAC-only nutrition programming. There is a consensus that MUAC is a better predictor of mortality than WFH [4–10] and it was reported that using MUAC alone is preferable for identifying high-risk malnourished children [11]. It is the most field-appropriate anthropometric measure, with the addition of the presence of bipedal oedema, to screen and detect cases of malnutrition in communities (4–9) and can be used by mothers ([12,13]. Throughout the paper, AM is based on MUAC assessment alone.

**Table 1:** Acute malnutrition definition and classification.

Case definition	
Severe Acute Malnutrition (SAM)	WFH < -3 SD *and/or* oedema *and/or* MUAC<115 mm
Global Acute Malnutrition (GAM)*	WFH < -2 SD *and/or* oedema *and/or* MUAC<125 mm

WFH: Weight-for-Height/Length; MUAC: Middle-Upper Arm Circumference

*WHO has not endorsed MUAC<125mm as being a measure of GAM but for the purpose of this study, MUAC<125mm will be referred to as GAM.

The assessment of the prevalence of acute malnutrition in children under five is widely used for the detection of nutritional emergencies, planning interventions, advocacy and programme monitoring and evaluation. Its estimation usually relies on cross-sectional cluster sample surveys [14–16] which are labour and resource intensive (i.e. time, logistics, and finance). Furthermore, surveys are not able to provide the frequency and geographic resolution of data that would enable swift detection and targeted response to crises before they are well-established [16–20].

**Fig 1:**
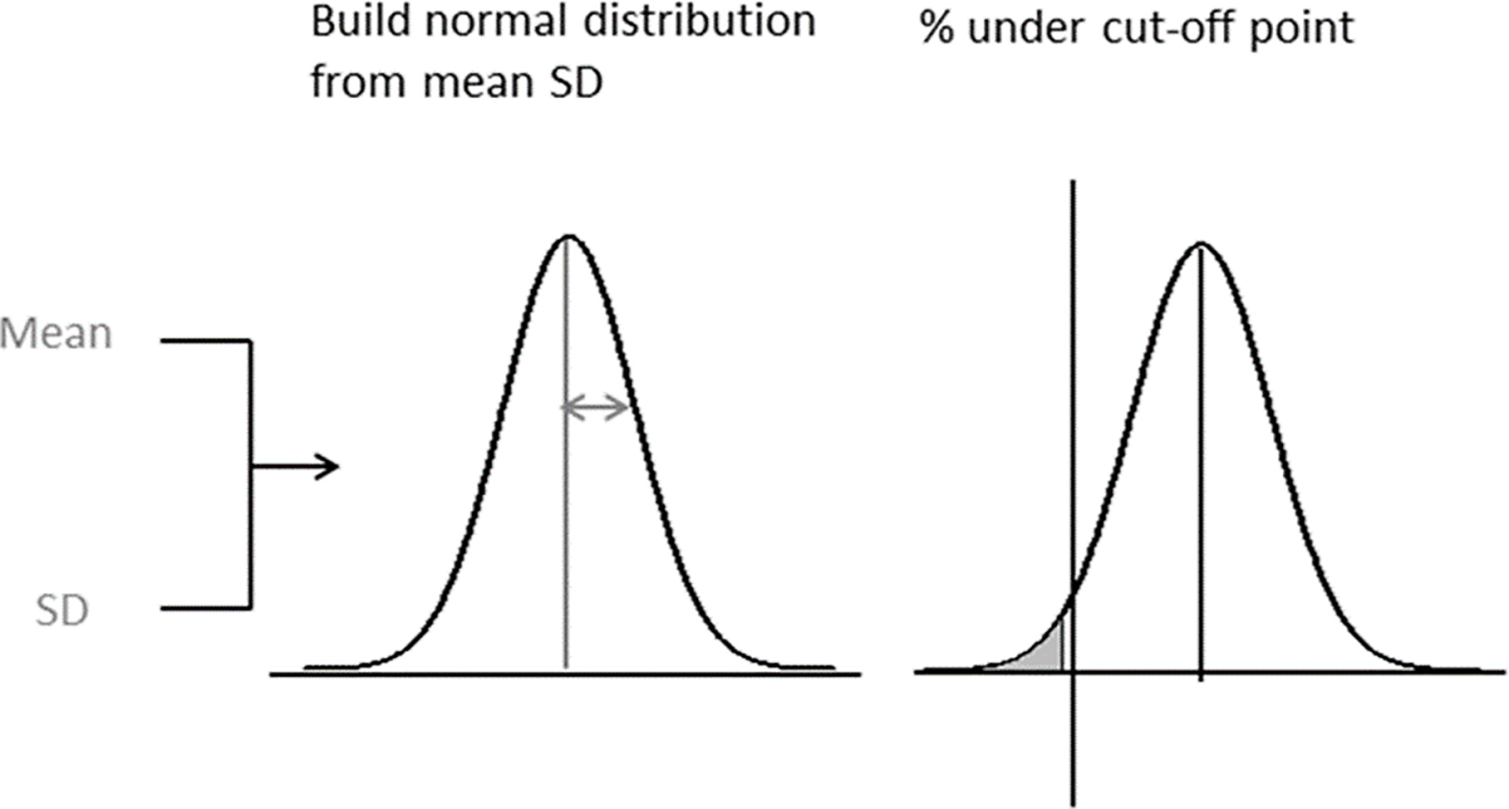
PROBIT method.

The PROBIT Method has been proposed as a more feasible alternative to standard surveys. This method estimates the prevalence of GAM according to any cut-off of interest by using the observed mean and standard deviation (SD) of anthropometric indices (e.g. MUAC) to construct a distribution, assumed to be normal in shape, and computing the percentage of the distribution that falls below the cut-off (see Fig 1)[21,22]. The method treats nutritional indices as continuous variables, instead of transforming each child observation into a binary datum (below or above cut-off), and as such has the possible advantage of decreasing the sample size required to estimate prevalence, while maintaining the same precision. Previous work has suggested that the assumption of a normal distribution is reasonable for MUAC, rendering the PROBIT approach potentially suitable for this index [23].

Previous studies have found that for simple random sample surveys, the PROBIT based prevalence estimate of acute malnutrition may have superior precision but can be subject to bias (Dale et al [22] and Blanton et al [24]). This study examined the use of the PROBIT Method for simple random samples and two-stage cluster samples to estimate SAM/GAM in children under five. We assessed two methods:

- PROBIT Method I, which takes the mean MUAC from the survey sample data and the MUAC SD from a database of previous surveys; and
- PROBIT Method II, which applies both the mean and SD of MUAC as observed in the survey sample.

We assessed the performance of both methods and the standard prevalence survey method (hereafter referred to as Classic Method) for estimation and classification purposes. To do so, we examined:

- the bias, precision and coverage (proportion of 95%CIs from the test method that contains the true prevalence value) of SAM and GAM prevalence estimates overall, by GAM category and by region
- the potential sources of bias of the tested methods
- the probability of correctly classifying GAM prevalence according to programmatically important thresholds (5%, 10% and 15%).

## Materials and methods

### Ethical statement

The project relied only on re-analysis of secondary data sources, none of which had uniquely identifiable information associated with each child-observation. Ethics approval for the project was sought and was obtained from the Ethics Committee of the London School of Hygiene and Tropical Medicine (LSHTM Ethics reference 6158)

### Data sources

**Fig 2:**
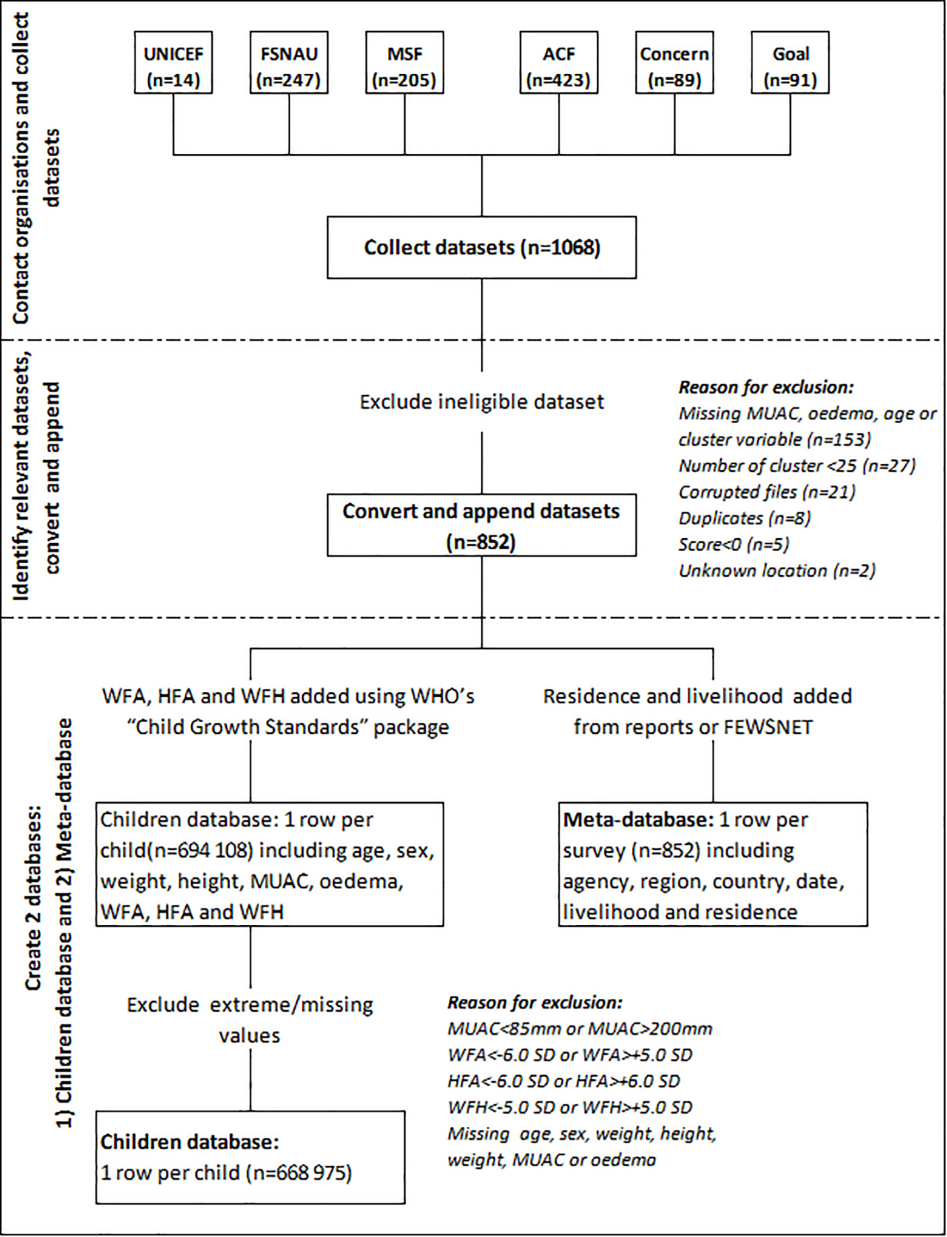
Data management.

The study relied entirely on previously collected survey data. Eligible datasets had to: (1) include anthropometric data: MUAC, oedema, age, weight and height as well as meta-data on country, livelihood, residence, cluster (if cluster surveys) and date; (2) have a minimum of 25 clusters if cluster surveys [25,26] (Fig 2).

Of the 1068 surveys collected, 852 surveys were included in this secondary data analysis (55 exhaustive surveys and 797 cluster sample surveys). The 852 surveys contained 694 108 children of which 25 134 presented highly improbable values and were excluded from the analysis (Fig 2).

### Simulation of small sample surveys

We tested the performance of PROBIT Method I, PROBIT Method II and the Classic Method, on simulated survey samples of varying size, drawn randomly from the larger survey database. Specifically, we generated sub-samples from each of the 852 survey datasets, simulating 100 surveys for each of eight different sample size (25, 50, 75, 100, 125, 150, 175, 200). To take into account the underlying clustered data structure when generating sub-samples from cluster survey datasets, we selected 25 clusters randomly, and, within each cluster, 1 to 8 child observations, again at random, to obtain sample sizes from 25 to 200. For non-cluster surveys, 25 to 200 children were randomly selected. Overall, the methods were tested on 681 600 (852 x 100 x 8) simulated surveys.

### Calculation of true prevalence

The prevalence point estimates were calculated from each of the 852 surveys and were taken as the true population prevalence (proportion of children with MUAC below 125mm for GAM and 115mm for SAM). This amounted to considering the source surveys as population-representative data.

### Implementation of each method

#### i) Classic method

For each simulated sample, the prevalence was calculated as the proportion of children with MUAC below 125mm for GAM and 115mm for SAM. Confidence intervals for the prevalence were calculated using cluster adjusted standard errors for the proportion.

#### ii) PROBIT method I–Mean given simulated sample and SD from previous surveys

For each simulated sample, the PROBIT function was used to calculate the prevalence as the cumulative probability of MUAC less than the cut-off of interest, given a normal distribution of MUAC. The mean MUAC used to parameterise this distribution was the mean MUAC in the simulated sample, while the standard deviation (SD) of MUAC was the MUAC SD from previous surveys.

The MUAC SD from previous surveys were weighted using effective sample size (25), stratified by region (in order to minimise MUAC SD variability) and bootstrapped with 2000 replications in each region. The mean MUAC SD from each bootstrap (for each region) was then used with mean MUAC in simulated sample to parameterise the normal distribution. The mean MUAC SD used were 12.3 mm for East Africa, 12.8 mm for West Africa, 13.0 mm for Central and South Africa, 12.8 mm for the Caribbean and 12.0mm for Asia.

The 95% confidence interval for the PROBIT prevalence was estimated using cluster bootstrapping with 2000 replications (2000 mean MUAC replications and 2000 weighted MUAC SD replications). For each replication the PROBIT Z score was calculated using MUAC SD randomly selected from the empirical distribution of MUAC SD of previous surveys in that geographic stratum and the mean MUAC from the bootstrap sample of the simulated survey. The standard error from the bootstrap distribution was used to calculate the confidence interval for the Z score, which was then transformed using the PROBIT function to calculate upper and lower confidence limits for the prevalence.

#### iii) PROBIT method II–Mean and SD from given simulated sample

For each simulated sample, the prevalence estimates were calculated using the PROBIT function to calculate the cumulative probability of MUAC less than the given threshold using mean MUAC and MUAC SD from the simulated sample. The same bootstrapping method as above was applied to compute confidence intervals using MUAC SD and mean MUAC from the bootstrap replications of the simulated survey.

### Estimation approach: bias, precision and coverage

For each of the methods, we examined bias, precision and coverage, for different GAM prevalence categories (<5%, 5–9%, 10–14%, ≥15%) and per region to investigate possible characteristics that would confound the outcome of the methods.

Bias was defined as the average difference between the estimated prevalence generated by each test method and the true prevalence.

Precision was defined as the average length of the 95%CIs generated by each test method (Abs [upper bound–lower bound] / 2).

Coverage was defined as the proportion of the 95%CIs from the test method that contained the true prevalence value. If coverage is as expected, the nominal 95% CI of the proposed methods should contain the true value 95% of the time.

To further assess possible characteristics that would influence bias, we used linear regression to explore associations between bias of GAM estimates as the dependent variable and the following independent variables: region, GAM categories based on MUAC (<5%, 5–9%, 10–14%, ≥15%), livelihood, residence, survey design (simple random sampling or clustered) and date (before/after 2006, 2006 corresponds to the adoption of the SMART Methodology [27]).

### Classification approach: probability of correctly classifying GAM prevalence

For each survey, the true GAM prevalence and the estimated GAM prevalence from the different methods were split into two categories according to different thresholds: GAM below 5%, 10% or 15% and GAM equal or above 5%, 10% or 15%. We then calculated the probability that the different methods correctly classify GAM prevalence ≥ threshold of interest.

## Results

### “True” prevalence observed in the database

The prevalence of GAM and SAM according to MUAC across surveys (n = 852) varied from 1% to 47.7% and from 0% to 20.6% respectively. Mean GAM and SAM were 9.9% and 2.2% respectively.

### Estimation approach

#### Bias

**Fig 3:**
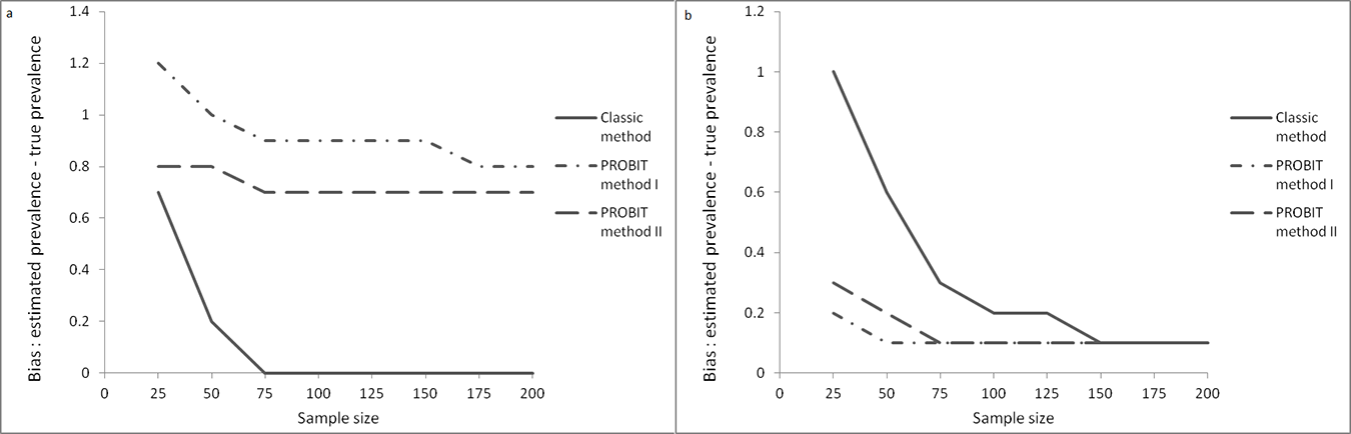
Bias in GAM (a) and SAM (b) estimates (%).

The mean bias in the estimations of GAM prevalence tended to be larger, the smaller the sample size. The estimates of GAM prevalence were practically unbiased using the classic method for sample size above 50. Both PROBIT Methods overestimated slightly the prevalence of GAM (Fig 3).

The bias in the estimation of SAM was minimal using PROBIT Methods while the Classic Method was biased for sample sizes under 75 (Fig 3). Individual simulated surveys showed both positive and negative bias (for SAM and GAM estimates) for all methods.

The bias of GAM and SAM varies by GAM category and by region. This analysis can be found in the S1 and S2 Tables.

#### Precision

**Fig 4:**
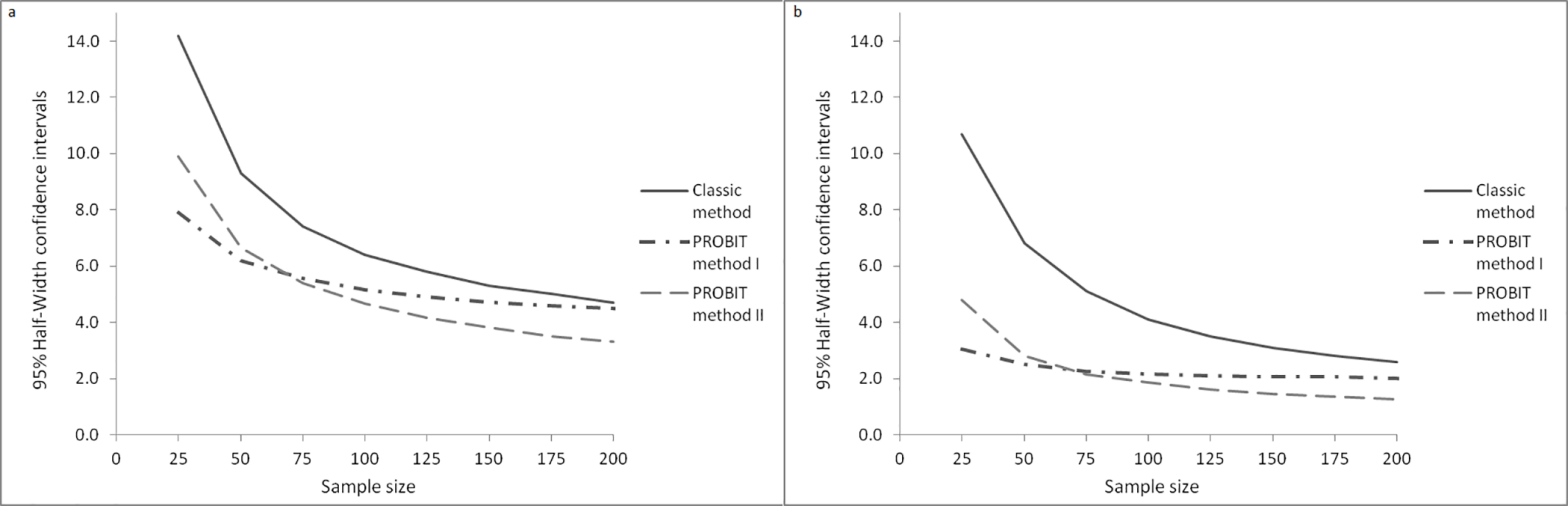
Precision of GAM (a) and SAM (b) estimates (%).

The precision of GAM and SAM prevalence estimates increased as the sample size increased for all methods. The classic method had the lowest precision for both GAM and SAM (see Fig 4).

For all methods, the precision of GAM and SAM estimates varies by GAM categories and by region. This analysis can be found in the S1 and S2 Tables.

#### Coverage

The PROBIT Methods had a clear advantage in term of coverage compared to the classic method for sample sizes < 50 for GAM and for sample sizes <150 for SAM. The classic method showed extremely low coverage of SAM estimates for small sample sizes, while coverage for both PROBIT methods was higher and more stable (see Table 2). For all methods, the coverage of GAM and SAM estimates varies by GAM categories and by region. This analysis can be found in the S1 and S2 Tables.

**Table 2:** Coverage of different methods for GAM and SAM.

	GAM			SAM		
Sample size	Classic method	PROBIT method I	PROBIT method II	Classic method	PROBIT method I	PROBIT method II
	(%)	(%)	(%)	(%)	(%)	(%)
25	83.7	92.1	94.5	35.4	89.9	92.7
50	93.9	91.2	93.6	55.3	88.8	91.1
75	96.5	90.4	93.1	67.1	88	89.7
100	97.3	89.8	92.5	75.2	87.4	88.7
125	98.1	89.4	91.8	80.4	87.3	87.7
150	98.4	88.9	91	84.2	87.1	86.5
175	98.4	88.8	90.4	87.3	87	85.7
200	98.6	88.4	89.9	89.6	86.8	84.5

#### Potential sources of bias

We investigated possible sources of bias by computing univariable linear regression with mean bias in GAM estimates as dependent variable and the following independent variable: region, GAM categories, livelihood, residence, survey design and date. The multivariable linear models built with variables showing univariable association are presented in Table 3 for the PROBIT Method I and the PROBIT Method II.

**Table 3:** Multivariable regression of mean bias in GAM estimates using the PROBIT Method I and PROBIT Method II.

	PROBIT Method I			PROBIT Method II		
Region	Coef	95% CI	P-value	Coef	95% CI	P-value
East Africa	-	-	-	-	-	-
Asia	0.431	0.400; 0.462	<0.001	0.239	0.210; 0.268	<0.001
Caribbean	-1.772	-1.832; -1.711	<0.001	-0.790	-0.847; -0.733	<0.001
C & S Africa	-0.094	-0.117; -0.071	<0.001	0.048	0.026; 0.069	<0.001
West Africa	-0.626	-0.650; -0.602	<0.001	-0.293	-0.316; -0.271	<0.001
**GAM (MUAC)**				** **		
<5%	-	-	-	-	-	-
5–9%	-0.185	-0.207; -0.164	<0.001	0.176	0.156; 0.196	<0.001
10–14%	-0.487	-0.511; -0.464	<0.001	0.311	0.288; 0.333	<0.001
≥ 15%	-0.464	-0.490; -0.438	<0.001	0.280	0.255; 0.304	<0.001
**Livelihood**						
Agriculture	-	-	-	-	-	-
Agro-Pastoral	0.454	0.435; 0.472	<0.001	0.179	0.161; 0.197	<0.001
Other	-0.466	-0.489; -0.443	<0.001	0.288	0.266; 0.309	<0.001
Pastoral	0.139	0.114; 0.163	<0.001	0.545	0.522; 0.568	<0.001
**Residence**						
Rural	-	-	-	-	-	-
Displaced	-1.801	-1.822; -1.779	<0.001	-0.572	-0.592; -0.552	<0.001
Other	-0.659	-0.683; -0.634	<0.001	-0.180	-0.203; -0.157	<0.001
Urban	-0.994	-1.022; -0.966	<0.001	-0.284	-0.311; -0.258	<0.001
**Sample size**						
25	-	-	-	-	-	-
50	-0.176	-0.204; -0.147	<0.001	-0.063	-0.090; -0.036	<0.001
75	-0.240	-0.269; -0.212	<0.001	-0.094	-0.121; -0.067	<0.001
100	-0.283	-0.312; -0.255	<0.001	-0.105	-0.132; -0.078	<0.001
125	-0.290	-0.318; '-0.261	<0.001	-0.109	-0.136; -0.082	<0.001
150	-0.303	-0.331; -0.274	<0.001	-0.111	-0.138; -0.084	<0.001
175	-0.321	-0.350; -0.293	<0.001	-0.110	-0.137; -0.083	<0.001
200	-0.324	-0.352; -0.295	<0.001	-0.114	-0.140; -0.087	<0.001
**Date**						
Before 2006	-	-	-	-	-	-
After 2006	0.691	0.675; 0.707	<0.001	0.026	0.011; 0.041	0.001
**Survey design**				** **		
Simple random	-	-	-	-	-	-
Clustered	-0.807	-0.839; -0.775	<0.001	-0.039	-0.069; -0.009	0.011

The differences observed by GAM category and by region are still apparent and statistically significant in the multivariable models for all methods (see Table 3).

### Classification approach

The probability of correctly identifying the GAM prevalence as above 5%, 10% or 15% decreased as the threshold used increased and increased as the sample size increased. The PROBIT Methods had better outcomes for smaller sample size compared to the Classic Method. The probability of correctly identifying the GAM prevalence as above 5% was high while for a 10% and 15% threshold, it was quite low (see Table 4).

**Table 4:** Probability of correctly classifying the true prevalence of GAM as exceeding a threshold of 5%, 10% or 15% GAM prevalence for the different methods.

Sample size	Probability of GAM ≥ 5% (%)			Probability of GAM ≥ 10% (%)			Probability of GAM ≥ 15% (%)		
	Classic method	PROBIT I	PROBIT II	Classic method	PROBIT I	PROBIT II	Classic method	PROBIT I	PROBIT II
25	87.7	91.2	92.4	56.0	69.7	69.6	32.7	55.7	52.1
50	91.4	91.5	93.4	67.1	72.8	74.8	53.1	62.4	61.4
75	92.7	91.7	93.7	77.8	74.2	77.4	66.5	65.9	66.8
100	93.2	91.6	94.0	78.8	75.0	79.3	68.1	68.0	69.8
125	95.8	91.8	94.3	84.0	75.4	80.6	73.2	68.6	70.9
150	95.7	91.7	94.4	83.7	75.8	81.4	77.4	69.4	72.6
175	95.5	91.8	94.5	86.9	75.9	82.1	81.0	70.1	73.7
200	95.5	91.8	94.7	86.2	76.1	82.3	79.3	70.6	74.5

## Discussion

This study analysed an exceptionally large database of anthropometric surveys conducted mostly in emergency situations, to test the performance of two candidate methods for acute malnutrition prevalence estimation, compared to the current mainstay method. Importantly, this study suggests that two PROBIT Methods, relying on far lower sample sizes that is typically the case with classic anthropometric surveys, had better performance than the Classic Method for estimating prevalence of GAM and SAM in a simulation of small surveys based on real survey data. The PROBIT Methods had better precision than the Classic Method for all sample sizes and a better coverage for smaller sample sizes, while having relatively little bias.

The mean bias in GAM estimates did not vary much between sample sizes and was slightly lower for PROBIT Method II (approximately 0.9 for PROBIT Method I and 0.7 for PROBIT Method II); it was very low for SAM estimates. Although the PROBIT methods overestimated the prevalence of GAM, individual simulated surveys showed both negative and positive bias. Therefore, it would not be possible to correct the bias by applying a systematic downward correction, as was suggest by Dale et al [22] who also found that the PROBIT method overestimated SAM/GAM. The mean bias for the Classic Method was positive for small sample sizes. However, this reflects the fact that the skewed distribution of the sample prevalence estimates lead to positive errors of greater magnitude than for the negative errors. As expected, the median bias for the Classic Method was close to zero at all sample sizes.

The precision of PROBIT Methods was systematically greater than the precision from the Classic Method. It was reasonable starting at a sample size of 50 for PROBIT Method I (6%) and from a sample size of 75 for PROBIT Method II (5.5%). Dale et al [22]found that the advantage in terms of precision of PROBIT versus the Classic Method is only applicable to sample sizes under 150. In theory, the precision of PROBIT methods should be higher regardless of the sample size. Blanton and Bilukha [22] had therefore explored the discrepancy between Dale et al. findings and theoretically expected results and found that the precision of PROBIT methods is superior for all sample size.

Blanton and Bilukha [22] had also concluded that bias from PROBIT method is population dependent, which is supported by our results. We found the PROBIT methods had smaller bias with higher level of GAM. The PROBIT methods also had minimal mean bias in the Caribbean and higher bias in Asia; different sample sizes might be required depending on the region. The PROBIT method also showed differences in bias between different livelihood zones, residence status and time period.

As expected, the precision of all methods depended on sample size and GAM prevalence, with worse precision as prevalence became closer to 50%, and with smaller sample size. However, precision was better for the PROBIT methods than the Classic method for all sample sizes.

The PROBIT Methods had better classification performance for smaller sample sizes for all cut-off points assessed. The 10% and 15% thresholds yield low probabilities of classifying the situation correctly while the 5% threshold classified the situation correctly over 90% of the time with a sample size as small as 25 for both PROBIT Methods.

Other strengths of our paper were:

1. It showed that PROBIT Methods can produce robust estimates for sample sizes as small as 50. This important finding opens up avenues for the use of either PROBIT method as part of nutrition surveillance systems and in particular for early warning, since the small sample size requirement would enable regular data collection and timely generation of information.
2. It examined the performance of the PROBIT Method in different regions: the sample size required may differ depending on the region.
3. It assessed the PROBIT Method for a range of survey designs and is the first to include analysis for clustered sample surveys.
4. It focuses on the assessment of AM with MUAC which is more feasible in the community and allows for rapid screening. The use of MUAC for community surveillance is best to assess the number of children in need of treatment as there is an increasing interest in MUAC-only nutrition programming (4–8)
5. It explored the possibility of using PROBIT with a classification approach.

We also recognise some limitations:

1. Surveys were used as proxies for true populations. Although it is hard to quantify the impact on the bias and precision of the proposed methods, we do not believe it would outweigh the advantages of the methods
2. We did not factor in oedematous malnutrition. However, estimates of GAM are generally similar whether including oedematous malnutrition or not (3), with the exception of the few areas with very high kwashiorkor burden.
3. We did not transform or smooth our data from the simulated sample to ensure the MUAC distribution approximated normality. Previous work has suggested that the assumption of a normal distribution is reasonable for MUAC. That study also showed that different transformations or smoothing techniques may be required for “non-normal” distributions to reach normality which would render this method more complicated(21). Furthermore, the bias in prevalence estimates was only very slightly reduced when Dale et al(18) assessed normal transformed data compared to non-transformed data. We therefore do not believe this significantly impacts our outcomes.
4. The design of the 852 surveys was taken into account when simulating samples but the design effect was not further factored in when calculating the confidence intervals of both PROBIT methods which may have underestimated the width of the confidence intervals for both methods.
5. Although the assessment of acute malnutrition is traditionally done by estimating the prevalence, it should be assessed using incidence (28, 29).

Future work could assess:

- Which of the two PROBIT methods is better for routine field use (e.g. considering usability, practicability). The PROBIT Method I requires previous surveys data from the region where the assessment is taking place. We may be able to use the SD MUAC used in the present work or use country level SD.
- Other approaches in the PROBIT estimation. There are other approaches that might be considered e.g. more non-parametric compared to the semi-parametric approach described here or a Bayesian approach to the PROBIT Method, incorporating the prior information from previous surveys as a way to potentially increase precision and decrease bias.
- User-friendly software packages / mobile phone platform where once raw-data entered, the results would be available immediately (e.g. ENA(30))

## Conclusion

Nutritional surveillance systems are critical for timely assessment of prevalence of acute malnutrition, enabling swift, well-targeted and effective responses. Our MUAC-based PROBIT methods have a clear advantage over classic methods. Their use would require much lower sample sizes which would enable great time and resource savings and thus would play a key role towards delivery and/or scale up of interventions.

## Supporting information

S1 Table Bias, precision and coverage of the different methods by wasting levels. (DOCX)

S2 Table Bias, precision and coverage of the different methods by region. (DOCX)
