# Supplementary material for: A novel, efficient method for estimating the prevalence of acute malnutrition in resource-constrained and crisis-affected settings: A simulation study
Source: PLoS One. 2017 Nov 1;12(11):e0186328. doi: 10.1371/journal.pone.0186328 (PMC5665500; doi:10.1371/journal.pone.0186328)
Supplement: S1 Table — (DOCX) [file pone.0186328.s001.docx]

**Supplemental file** **1: Bias, precision and coverage of the different methods by wasting levels**

Table 1: Bias of wasting (a, b, c) and severe wasting (d, e, f) estimates by wasting categories (based on MUAC) (%)

| (a) Classic Method | (b) Probit Method I | (c) Probit Method II |
| --- | --- | --- |
| \| Sample size \| Wasting \| \| \| \| \| \| --- \| --- \| --- \| --- \| --- \| --- \| \| All \| <5% \| 5-9% \| 10-14% \| ≥15% \| \| Mean (%) \| Mean (%) \| Mean (%) \| Mean (%) \| Mean (%) \| \| 25 \| 0.7 \| 1.3 \| 1.0 \| 0.4 \| 0.0 \| \| 50 \| 0.2 \| 0.5 \| 0.1 \| 0.1 \| 0.0 \| \| 75 \| 0.0 \| 0.2 \| 0.0 \| 0.0 \| 0.0 \| \| 100 \| 0.0 \| 0.1 \| 0.0 \| 0.0 \| 0.1 \| \| 125 \| 0.0 \| 0.0 \| 0.0 \| 0.0 \| 0.0 \| \| 150 \| 0.0 \| 0.0 \| 0.0 \| 0.0 \| 0.0 \| \| 175 \| 0.0 \| 0.0 \| 0.0 \| 0.0 \| 0.0 \| \| 200 \| 0.0 \| 0.0 \| 0.0 \| 0.0 \| 0.0 \| | \| Sample size \| Wasting \| \| \| \| \| \| --- \| --- \| --- \| --- \| --- \| --- \| \| All \| <5% \| 5-9% \| 10-14% \| ≥15% \| \| Mean (%) \| Mean (%) \| Mean (%) \| Mean (%) \| Mean (%) \| \| 25 \| 1.2 \| 1.4 \| 1.3 \| 0.9 \| 1.1 \| \| 50 \| 1.0 \| 1.2 \| 1.1 \| 0.7 \| 0.9 \| \| 75 \| 0.9 \| 1.2 \| 1.0 \| 0.6 \| 0.8 \| \| 100 \| 0.9 \| 1.2 \| 1.0 \| 0.6 \| 0.7 \| \| 125 \| 0.9 \| 1.2 \| 1.0 \| 0.5 \| 0.7 \| \| 150 \| 0.9 \| 1.1 \| 1.0 \| 0.6 \| 0.7 \| \| 175 \| 0.8 \| 1.1 \| 1.0 \| 0.5 \| 0.7 \| \| 200 \| 0.8 \| 1.1 \| 1.0 \| 0.5 \| 0.7 \| | \| Sample size \| Wasting \| \| \| \| \| \| --- \| --- \| --- \| --- \| --- \| --- \| \| All \| <5% \| 5-9% \| 10-14% \| ≥15% \| \| Mean (%) \| Mean (%) \| Mean (%) \| Mean (%) \| Mean (%) \| \| 25 \| 0.8 \| 0.8 \| 0.9 \| 0.9 \| 0.7 \| \| 50 \| 0.8 \| 0.6 \| 0.8 \| 0.9 \| 0.8 \| \| 75 \| 0.7 \| 0.5 \| 0.7 \| 0.8 \| 0.9 \| \| 100 \| 0.7 \| 0.5 \| 0.7 \| 0.8 \| 0.9 \| \| 125 \| 0.7 \| 0.5 \| 0.7 \| 0.8 \| 0.9 \| \| 150 \| 0.7 \| 0.5 \| 0.7 \| 0.8 \| 0.9 \| \| 175 \| 0.7 \| 0.5 \| 0.7 \| 0.8 \| 0.9 \| \| 200 \| 0.7 \| 0.5 \| 0.7 \| 0.8 \| 0.9 \| |
| (d) Classic Method | (e) Probit Method I | (f) Probit Method II |
| \| Sample size \| Severe wasting \| \| \| \| \| \| --- \| --- \| --- \| --- \| --- \| --- \| \| All \| <5% \| 5-9% \| 10-14% \| ≥15% \| \| Mean (%) \| Mean (%) \| Mean (%) \| Mean (%) \| Mean (%) \| \| 25 \| 1.0 \| 0.5 \| 0.9 \| 1.3 \| 1.3 \| \| 50 \| 0.5 \| 0.4 \| 0.6 \| 0.6 \| 0.4 \| \| 75 \| 0.3 \| 0.3 \| 0.4 \| 0.3 \| 0.2 \| \| 100 \| 0.2 \| 0.3 \| 0.3 \| 0.2 \| 0.0 \| \| 125 \| 0.2 \| 0.2 \| 0.2 \| 0.1 \| 0.1 \| \| 150 \| 0.1 \| 0.2 \| 0.1 \| 0.1 \| 0.0 \| \| 175 \| 0.1 \| 0.1 \| 0.1 \| 0.1 \| 0.0 \| \| 200 \| 0.1 \| 0.1 \| 0.1 \| 0.0 \| 0.0 \| | \| Sample size \| Severe wasting \| \| \| \| \| \| --- \| --- \| --- \| --- \| --- \| --- \| \| All \| <5% \| 5-9% \| 10-14% \| ≥15% \| \| Mean (%) \| Mean (%) \| Mean (%) \| Mean (%) \| Mean (%) \| \| 25 \| 0.2 \| 0.1 \| 0.2 \| 0.2 \| 0.5 \| \| 50 \| 0.1 \| 0.0 \| 0.1 \| 0.1 \| 0.3 \| \| 75 \| 0.1 \| 0.0 \| 0.1 \| 0.1 \| 0.3 \| \| 100 \| 0.1 \| 0.1 \| 0.1 \| 0.0 \| 0.2 \| \| 125 \| 0.1 \| 0.1 \| 0.1 \| 0.0 \| 0.2 \| \| 150 \| 0.1 \| 0.1 \| 0.0 \| 0.0 \| 0.2 \| \| 175 \| 0.1 \| 0.1 \| 0.0 \| 0.0 \| 0.2 \| \| 200 \| 0.1 \| 0.1 \| 0.0 \| 0.0 \| 0.2 \| | \| Sample size \| Severe wasting \| \| \| \| \| \| --- \| --- \| --- \| --- \| --- \| --- \| \| All \| <5% \| 5-9% \| 10-14% \| ≥15% \| \| Mean (%) \| Mean (%) \| Mean (%) \| Mean (%) \| Mean (%) \| \| 25 \| 0.3 \| 0.1 \| 0.2 \| 0.4 \| 0.6 \| \| 50 \| 0.2 \| 0.0 \| 0.1 \| 0.3 \| 0.5 \| \| 75 \| 0.1 \| 0.0 \| 0.0 \| 0.2 \| 0.5 \| \| 100 \| 0.1 \| 0.1 \| 0.0 \| 0.2 \| 0.4 \| \| 125 \| 0.1 \| 0.1 \| 0.0 \| 0.1 \| 0.4 \| \| 150 \| 0.1 \| -0.1 \| 0.0 \| 0.1 \| 0.4 \| \| 175 \| 0.1 \| -0.1 \| 0.0 \| 0.1 \| 0.4 \| \| 200 \| 0.1 \| -0.1 \| 0.0 \| 0.1 \| 0.4 \| |

Table 2: Precision (Half 95% CI) of wasting (a, b, c) and severe wasting (d, e, f) estimates by wasting categories(based on MUAC) (%)

| (a) Classic Method | (b) Probit Method I | (c) Probit Method II |
| --- | --- | --- |
| \| Sample size \| Wasting \| \| \| \| \| \| --- \| --- \| --- \| --- \| --- \| --- \| \| All \| <5% \| 5-9 % \| 10-14 % \| ≥15% \| \| Mean (%) \| Mean  (%) \| Mean  (%) \| Mean  (%) \| Mean  (%) \| \| 25 \| 14.2 \| 11.7 \| 13.6 \| 14.9 \| 16.5 \| \| 50 \| 9.3 \| 7.3 \| 8.6 \| 10.1 \| 11.7 \| \| 75 \| 7.4 \| 5.5 \| 6.8 \| 8.2 \| 9.7 \| \| 100 \| 6.4 \| 4.6 \| 5.8 \| 7.2 \| 8.6 \| \| 125 \| 5.8 \| 4.0 \| 5.2 \| 6.5 \| 7.9 \| \| 150 \| 5.3 \| 3.6 \| 4.8 \| 6.0 \| 7.4 \| \| 175 \| 5.0 \| 3.3 \| 4.5 \| 5.6 \| 6.9 \| \| 200 \| 4.7 \| 3.1 \| 4.2 \| 5.3 \| 6.6 \| | \| Sample size \| Wasting \| \| \| \| \| \| --- \| --- \| --- \| --- \| --- \| --- \| \| All \| <5% \| 5-9 % \| 10-14 % \| ≥15% \| \| Mean (%) \| Mean  (%) \| Mean  (%) \| Mean  (%) \| Mean  (%) \| \| 25 \| 7.9 \| 4.9 \| 7.0 \| 9.1 \| 11.4 \| \| 50 \| 6.2 \| 3.9 \| 5.6 \| 7.1 \| 8.7 \| \| 75 \| 5.5 \| 3.6 \| 5.1 \| 6.3 \| 7.6 \| \| 100 \| 5.1 \| 3.4 \| 4.7 \| 5.8 \| 6.9 \| \| 125 \| 4.9 \| 3.3 \| 4.6 \| 5.5 \| 6.4 \| \| 150 \| 4.7 \| 3.2 \| 4.4 \| 5.3 \| 6.1 \| \| 175 \| 4.6 \| 3.2 \| 4.3 \| 5.2 \| 5.9 \| \| 200 \| 4.5 \| 3.1 \| 4.3 \| 5.1 \| 5.7 \| | \| Sample size \| Wasting \| \| \| \| \| \| --- \| --- \| --- \| --- \| --- \| --- \| \| All \| <5% \| 5-9 % \| 10-14 % \| ≥15% \| \| Mean (%) \| Mean  (%) \| Mean  (%) \| Mean  (%) \| Mean  (%) \| \| 25 \| 9.9 \| 6.6 \| 9.2 \| 11.2 \| 13.0 \| \| 50 \| 6.7 \| 4.2 \| 6.1 \| 7.7 \| 9.0 \| \| 75 \| 5.4 \| 3.3 \| 4.9 \| 6.2 \| 7.4 \| \| 100 \| 4.6 \| 2.9 \| 4.2 \| 5.4 \| 6.4 \| \| 125 \| 4.2 \| 2.5 \| 3.8 \| 4.8 \| 5.7 \| \| 150 \| 3.8 \| 2.3 \| 3.5 \| 4.4 \| 5.2 \| \| 175 \| 3.5 \| 2.1 \| 3.2 \| 4.1 \| 4.9 \| \| 200 \| 3.3 \| 2.0 \| 3.0 \| 3.8 \| 4.6 \| |
| (d) Classic Method | (e) Probit Method I | (f) Probit Method II |
| \| Sample size \| Severe wasting \| \| \| \| \| \| --- \| --- \| --- \| --- \| --- \| --- \| \| All \| <5% \| 5-9 % \| 10-14 % \| ≥15% \| \| Mean (%) \| Mean  (%) \| Mean  (%) \| Mean  (%) \| Mean  (%) \| \| 25 \| 10.7 \| 6.0 \| 9.7 \| 11.7 \| 12.9 \| \| 50 \| 6.8 \| 4.5 \| 6.4 \| 7.2 \| 8.0 \| \| 75 \| 5.1 \| 3.6 \| 4.7 \| 5.4 \| 6.1 \| \| 100 \| 4.1 \| 3.0 \| 3.8 \| 4.4 \| 5.1 \| \| 125 \| 3.5 \| 2.6 \| 3.2 \| 3.7 \| 4.5 \| \| 150 \| 3.1 \| 2.2 \| 2.8 \| 3.3 \| 4.1 \| \| 175 \| 2.8 \| 2.0 \| 2.5 \| 3.0 \| 3.8 \| \| 200 \| 2.6 \| 1.8 \| 2.3 \| 2.8 \| 3.6 \| | \| Sample size \| Severe wasting \| \| \| \| \| \| --- \| --- \| --- \| --- \| --- \| --- \| \| All \| <5% \| 5-9 % \| 10-14 % \| ≥15% \| \| Mean (%) \| Mean  (%) \| Mean  (%) \| Mean  (%) \| Mean  (%) \| \| 25 \| 3.0 \| 1.4 \| 2.4 \| 3.6 \| 5.5 \| \| 50 \| 2.5 \| 1.2 \| 2.0 \| 2.9 \| 4.4 \| \| 75 \| 2.3 \| 1.1 \| 1.8 \| 2.7 \| 4.0 \| \| 100 \| 2.2 \| 1.0 \| 1.8 \| 2.6 \| 3.8 \| \| 125 \| 2.1 \| 1.0 \| 1.7 \| 2.5 \| 3.6 \| \| 150 \| 2.1 \| 1.0 \| 1.7 \| 2.4 \| 3.6 \| \| 175 \| 2.0 \| 1.0 \| 1.7 \| 2.4 \| 3.5 \| \| 200 \| 2.0 \| 1.0 \| 1.6 \| 2.4 \| 3.4 \| | \| Sample size \| Severe wasting \| \| \| \| \| \| --- \| --- \| --- \| --- \| --- \| --- \| \| All \| <5% \| 5-9 % \| 10-14 % \| ≥15% \| \| Mean (%) \| Mean  (%) \| Mean  (%) \| Mean  (%) \| Mean  (%) \| \| 25 \| 4.8 \| 2.6 \| 4.1 \| 5.7 \| 7.3 \| \| 50 \| 2.8 \| 1.3 \| 2.3 \| 3.4 \| 4.7 \| \| 75 \| 2.2 \| 0.9 \| 1.7 \| 2.7 \| 3.8 \| \| 100 \| 1.9 \| 0.8 \| 1.5 \| 2.3 \| 3.2 \| \| 125 \| 1.6 \| 0.6 \| 1.3 \| 2.0 \| 2.9 \| \| 150 \| 1.5 \| 0.6 \| 1.1 \| 1.8 \| 2.6 \| \| 175 \| 1.4 \| 0.5 \| 1.0 \| 1.7 \| 2.4 \| \| 200 \| 1.3 \| 0.5 \| 1.0 \| 1.6 \| 2.3 \| |

Table 3: Coverage of wasting (a, b, c) and severe wasting (d, e, f) estimates by wasting categories (based on MUAC) (%)

| (a) Classic Method | (b) Probit Method I | (c) Probit Method II |
| --- | --- | --- |
| \| Sample size \| Wasting \| \| \| \| \| \| --- \| --- \| --- \| --- \| --- \| --- \| \| All  (%) \| <5%  (%) \| 5-9  (%) \| 10-14%  (%) \| ≥15%  (%) \| \| 25 \| 83.7 \| 56.9 \| 82.7 \| 94.4 \| 97.8 \| \| 50 \| 93.9 \| 80.6 \| 95.4 \| 97.9 \| 97.9 \| \| 75 \| 96.5 \| 90.2 \| 97.8 \| 97.8 \| 97.6 \| \| 100 \| 97.3 \| 94.2 \| 98.2 \| 97.5 \| 98.0 \| \| 125 \| 97.8 \| 96.3 \| 98.2 \| 97.9 \| 98.3 \| \| 150 \| 98.1 \| 97.8 \| 98.2 \| 98.0 \| 98.4 \| \| 175 \| 98.4 \| 98.2 \| 98.4 \| 98.3 \| 98.6 \| \| 200 \| 98.6 \| 98.6 \| 98.6 \| 98.4 \| 98.8 \| | \| Sample size \| Wasting \| \| \| \| \| \| --- \| --- \| --- \| --- \| --- \| --- \| \| All  (%) \| <5%  (%) \| 5-9  (%) \| 10-14  (%) \| ≥15%  (%) \| \| 25 \| 92.1 \| 89.7 \| 92.1 \| 93.0 \| 93.0 \| \| 50 \| 91.2 \| 88.4 \| 91.4 \| 92.3 \| 92.2 \| \| 75 \| 90.4 \| 87.1 \| 90.6 \| 91.9 \| 90.9 \| \| 100 \| 89.8 \| 86.8 \| 90.2 \| 90.9 \| 90.2 \| \| 125 \| 89.4 \| 86.3 \| 89.7 \| 90.4 \| 89.6 \| \| 150 \| 88.9 \| 86.1 \| 89.5 \| 89.5 \| 88.9 \| \| 175 \| 88.8 \| 86.0 \| 89.4 \| 89.8 \| 88.5 \| \| 200 \| 88.4 \| 85.6 \| 89.2 \| 89.1 \| 87.9 \| | \| Sample size \| Wasting \| \| \| \| \| \| --- \| --- \| --- \| --- \| --- \| --- \| \| All  (%) \| <5%  (%) \| 5-9  (%) \| 10-14%  (%) \| ≥15%  (%) \| \| 25 \| 94.5 \| 93.7 \| 94.3 \| 94.9 \| 95.5 \| \| 50 \| 93.6 \| 92.7 \| 93.2 \| 93.8 \| 95.0 \| \| 75 \| 93.1 \| 91.4 \| 93.0 \| 93.7 \| 94.1 \| \| 100 \| 92.5 \| 90.8 \| 92.2 \| 93.2 \| 93.8 \| \| 125 \| 91.8 \| 89.9 \| 91.6 \| 92.4 \| 93.6 \| \| 150 \| 91.0 \| 88.6 \| 90.7 \| 91.8 \| 93.0 \| \| 175 \| 90.4 \| 88.1 \| 90.0 \| 91.4 \| 92.2 \| \| 200 \| 89.9 \| 87.5 \| 89.5 \| 90.8 \| 91.9 \| |
| (d) Classic Method | (e) Probit Method I | (f) Probit Method II |
| \| Sample size \| Severe wasting \| \| \| \| \| \| --- \| --- \| --- \| --- \| --- \| --- \| \| All  (%) \| <5%  (%) \| 5-9  (%) \| 10-14%  (%) \| ≥15%  (%) \| \| 25 \| 35.4 \| 10.4 \| 26.7 \| 46.5 \| 66.4 \| \| 50 \| 55.3 \| 22.4 \| 47.8 \| 70.2 \| 85.8 \| \| 75 \| 67.1 \| 33.1 \| 61.5 \| 82.6 \| 93.3 \| \| 100 \| 75.2 \| 42.5 \| 71.8 \| 89.0 \| 96.6 \| \| 125 \| 80.4 \| 49.9 \| 78.4 \| 93.2 \| 97.5 \| \| 150 \| 84.2 \| 55.9 \| 83.6 \| 95.2 \| 98.4 \| \| 175 \| 87.3 \| 61.9 \| 87.7 \| 96.5 \| 98.6 \| \| 200 \| 89.6 \| 66.8 \| 90.7 \| 97.4 \| 98.7 \| | \| Sample size \| Severe wasting \| \| \| \| \| \| --- \| --- \| --- \| --- \| --- \| --- \| \| All  (%) \| <5%  (%) \| 5-9%  (%) \| 10-14%  (%) \| ≥15%  (%) \| \| 25 \| 89.9 \| 86.5 \| 91.2 \| 91.2 \| 88.5 \| \| 50 \| 88.8 \| 85.1 \| 90.8 \| 89.8 \| 86.2 \| \| 75 \| 88.0 \| 83.9 \| 90.2 \| 89.3 \| 84.8 \| \| 100 \| 87.4 \| 83.7 \| 89.9 \| 88.3 \| 83.7 \| \| 125 \| 87.3 \| 83.2 \| 89.8 \| 88.3 \| 83.5 \| \| 150 \| 87.1 \| 82.7 \| 89.9 \| 87.8 \| 83.5 \| \| 175 \| 87.0 \| 82.5 \| 89.8 \| 87.8 \| 83.2 \| \| 200 \| 86.8 \| 82.4 \| 89.8 \| 87.6 \| 82.7 \| | \| Sample size \| Severe wasting \| \| \| \| \| \| --- \| --- \| --- \| --- \| --- \| --- \| \| All  (%) \| <5%  (%) \| 5-9  (%) \| 10-14%  (%) \| ≥15%  (%) \| \| 25 \| 92.7 \| 89.1 \| 93.2 \| 93.7 \| 93.7 \| \| 50 \| 91.1 \| 86.5 \| 91.7 \| 92.1 \| 92.6 \| \| 75 \| 89.7 \| 84.3 \| 90.4 \| 91.4 \| 90.8 \| \| 100 \| 88.7 \| 83.1 \| 89.6 \| 90.2 \| 89.7 \| \| 125 \| 87.7 \| 81.9 \| 88.7 \| 89.6 \| 88.5 \| \| 150 \| 86.5 \| 80.5 \| 87.5 \| 88.4 \| 87.3 \| \| 175 \| 85.7 \| 79.0 \| 86.7 \| 87.9 \| 86.4 \| \| 200 \| 84.5 \| 77.4 \| 85.6 \| 87.3 \| 85.0 \| |
